# Pregnancy-related factors induce immune tolerance through regulation of sCD83 release

**DOI:** 10.3389/fimmu.2024.1452879

**Published:** 2024-09-12

**Authors:** Pauline Krupa, Hannah Wein, Lea Sophie Zemmrich, Marek Zygmunt, Damián Oscar Muzzio

**Affiliations:** Research Laboratory, Department of Obstetrics and Gynecology, University Medicine Greifswald, Greifswald, Germany

**Keywords:** pregnancy, tolerance, CD83, lipopolysaccharide, hormones

## Abstract

A well-balanced maternal immune system is crucial to maintain fetal tolerance in case of infections during pregnancy. Immune adaptations include an increased secretion of soluble mediators to protect the semi-allogeneic fetus from excessive pro-inflammatory response. B lymphocytes acquire a higher capacity to express CD83 and secrete soluble CD83 (sCD83) upon exposure to bacteria-derived components such as LPS. CD83 possesses immune modulatory functions and shows a promising therapeutic potential against inflammatory conditions. The administration of sCD83 to pregnant mice reduces LPS-induced abortion rates. The increased CD83 expression by endometrial B cells as compared to peripheral blood B cells suggests its modulatory role in the fetal tolerance, especially in the context of infection. We postulate that in pregnancy, CD83 expression and release is controlled by pregnancy-related hormones. The intra- and extracellular expression of CD83 in leukocytes from peripheral blood or *decidua basalis* and *parietalis* at term were analyzed by flow cytometry. After treatment with pregnancy-related hormones and LPS, ELISA and qPCR were performed to study sCD83 release and *CD83* gene expression, respectively. Cleavage prediction analysis was used to find potential proteases targeting CD83. Expression of selected proteases was analyzed by ELISA. Higher levels of CD83 were found in CD11c^+^ dendritic cells, CD3^+^ T cells and CD19^+^ B cells from *decidua basalis* and *decidua parietalis* after LPS-stimulation *in vitro*. An increase of intracellular expression of CD83 was also detected in CD19^+^ B cells from both compartments. Stimulated B cells displayed significantly higher percentages of CD83^+^ cells than dendritic cells and T cells from *decidua basalis* and peripheral blood. Treatment of B lymphocytes with pregnancy-related molecules (E2, P4, TGF-β1 and hCG) enhanced the LPS-mediated increase of CD83 expression, while dexamethasone led to a reduction. Similarly, the release of sCD83 was increased under TGF-β1 treatment but decreased upon dexamethasone stimulation. Finally, we found that the hormonal regulation of CD83 expression is likely a result from a balance between gene transcription from *CD83* and the modulation of the metalloproteinase MMP-7. Thus, data supports and complements our previous murine studies on hormonal regulation of CD83 expression, reinforcing its immunomodulatory relevance in anti-bacterial responses during pregnancy.

## 1 Introduction

The dynamic balance between pro- and anti-inflammatory mediators is essential for the establishment of pregnancy, its progression as well as for the onset of parturition (1–4). The immune system undergoes multiple adaptations that facilitate the accommodation of the fetus, foster tolerance against paternal antigens and adjust defense strategies to protect mother and fetus against pathogens (5–10). Still, the presence of pathogens can lead to excessive immune responses, breaking the delicate immune balance and resulting in pregnancy complications including preterm birth (2).

The immune system possesses several mechanisms to control such excessive immune responses, promoting tolerance and the maintenance of pregnancy (11–13). In previous studies, we observed that pregnant mice showed a reduced antigen presentation potential, a distinct immunoglobulin and cytokine secretory milieu and an improved capacity to secrete anti-inflammatory mediators, including soluble CD83 (sCD83) (14–17).

CD83 is a transmembrane glycoprotein that belongs to the immunoglobulin family (18). Originally described as a maturation marker for dendritic cells (DCs), CD83 expression has been detected in other immune cells including T and B cells, where immune modulatory functions have been observed (19–21). In DCs and B cells, CD83 stabilizes the major histocompatibility molecule type II (MHC-II) through negative regulation of the E3 ubiquitin-protein ligases MARCH1 and MARCH8 (22, 23). In CD4^+^ T cells, CD83 expression is associated with the promotion of a tolerogenic phenotype (24, 25).

Murine experiments permitted a vast characterization of CD83 on B cells. Uhde et al. showed that CD83 co-localizes with the B cell receptor (BCR) and the Toll-like receptor 4 (TLR4) (26). The expression is associated with a lower sensitivity of the BCR and an increase in marginal zone (MZ) B cell number (26, 27). Furthermore, CD83 overexpression improved the release of the anti-inflammatory cytokine IL-10 in lipopolysaccharide (LPS) stimulated lymphocytes (28).

CD83 has a soluble form, sCD83, which possesses strong anti-inflammatory functions as successfully evaluated in mouse models for autoimmunity, allograft transplantation, allergies and chronic inflammation (29–36). In this context, we were able to show that mice acquire a higher capacity to release sCD83 during pregnancy and that lower sCD83 serum levels are associated with disturbed murine pregnancies (15, 17). Additionally, it has recently been shown that sCD83 can protect pregnant mice from LPS-mediated fetal death (37). One major immunomodulatory mechanism of sCD83 concerns the blockade of LPS-TLR4/MD-2 interactions (38). These data point to a regulation of immune responses through sCD83 upon confrontation with bacteria, which could contribute to maintenance of pregnancy.

Although CD83 expression has been described in human endometrial B cells, its role during pregnancy is less known (39). A recent study described the levels of circulating sCD83 in human pregnancies (40). Analogously to our observations in mice, hormonal regulation of serum sCD83 levels is suggested for human pregnancies as well. The aim of this work was to evaluate the regulation and expression of CD83 in human pregnancies. We postulate that local and systemic pregnancy associated factors regulate CD83 expression and sCD83 release.

## 2 Materials and methods

### 2.1 Isolation of PBMCs (CD3^+^ and CD19^+^ cells)

Buffy coats from non-pregnant female donors at reproductive age (18-40 years) were obtained from the Institute of Transfusion Medicine of the University Medicine Greifswald with approval of the Ethics Committee of the University of Greifswald (Reg.Nr. BB 014/14). At the time of blood donation, all women felt healthy, were at least 4 weeks absent of febrile infectious disease, antibiotics or systemic corticosteroids intake, and had not been pregnant for the last 6 months. PBMCs were isolated using density centrifugation. Buffy coats were centrifuged at 1300 × *g* for 10 min without brake. The pellets were resuspended in PBS (Biochrom AG, Berlin, D) and layered on Lymphoprep™ (STEMCELL technologies Inc, Vancouver, Canada). After centrifugation at 800 × *g* for 30 min without brake and acceleration, mononuclear cells were collected, washed in PBS and centrifuged at 300 × *g* for 5 min.

CD3^+^ and CD19^+^ cells were isolated using the EasySep™ Human CD3 Positive Selection Kit II (STEMCELL technologies Inc, Vancouver, Canada) and the CD19 MicroBeads, human Kit (Miltenyi Biotec GmbH, Bergisch Gladbach, D) following manufacturer’s instructions.

### 2.2 Isolation of decidual mononuclear cells

Decidual mononuclear cells were isolated from decidua basalis and parietalis from term placentae (38-41 weeks) after caesarean section according to Xu et al., 2015. Experiments have been approved by the Ethic Committee of the University of Greifswald (BB 160/17a). Women included in this study did neither have diagnosed immunological nor acute or chronic inflammatory diseases. Their age ranged between 22-42. More detailed information can be found in **Supplementary Table 1**. Manually scraped tissue was enzymatically digested using collagenase D (1.67 mg/mL) (Merck, Darmstadt, D) and DNase I, Bovine Pankreas (0.5 mg/mL) (Merck, Darmstadt, D). Mechanical digestion was performed using the gentleMACS(TM) Dissociator system (program ‘m_heart_02’) (Miltenyi Biotec GmbH, Bergisch Gladbach, D). Afterwards the tubes were incubated for 60 min at 37°C and mixed regularly. To gather the cells, the suspension was filtered using a 0.4 µm cell strainer and centrifuged and 350 × *g* for 10 min. PBMC isolation was performed according to 5.1.

### 2.3 Cell culture

PBMCs from buffy coats and decidual tissue as well as CD3^+^ and CD19^+^ cells were cultured in RPMI 1640 (10% FBS, 1% PenStrep, 55 μM β-ME) media (Biochrome GmbH, Berlin, D). 1 × 10^6^/mL PBMCs were cultured and stimulated on 24 well flat bottom suspension plates (Sarstedt AG&Co., Nümbrecht, D). For CD3^+^ and CD19^+^ cells, 4 × 10^6^ cells/mL were seeded in 48 well flat bottom suspension plates (Sarstedt AG&Co., Nümbrecht, D). The PBMCs from buffy coat, CD3^+^ and CD19^+^ cells were stimulated with LPS (10 μg/mL)(Merck, Darmstadt, D) and additionally treated with human chorionic gonadotrophin (hCG) (100 UI/mL) (Merck, Darmstadt, D), TGF-β1 (20 ng/mL), (Bio-Techne, Minneapolis, USA), estradiol (20 ng/mL) (Merck, Darmstadt, D), progesterone (300 ng/mL) (Merck, Darmstadt, D) or 1 μM dexamethasone (400 ng/mL) (Merck, Darmstadt, D) for 48h. For the last 5 h PMA (50 ng/mL) (Merck, Darmstadt, D) and Ionomycin (500 ng/mL) (Merck, Darmstadt, D) were added. PBMCs from decidual tissue were stimulated with 10 μg/mL LPS for 43 h before 50 ng/mL PMA and 500 n/mL Ionomycin were added for additional 5 h.

### 2.4 Isolation of extracellular vesicles

For downstream analysis using flow cytometry, extracellular vesicles (EVs) were isolated from PBMC supernatant after 48 h stimulation using Total Exosome Isolation reagent (Invitrogen, Thermo Fisher Scientific, Waltham, Massachusetts, USA), following manual instructions. For CD83 ELISA, EVs were isolated from PBMC supernatant using EV Isolation Kit Pan (Miltenyi Biotec GmbH, Bergisch Gladbach, D), following manufacturer’s instructions.

### 2.5 Flow cytometry

After 48 h of cultivation, cells were washed in PBS and stained with 100 μl Fixable Viability dye eFlour 520 (1/1.000) (Thermo Fisher Scientific, Waltham, Massachusetts, USA) in PBS for live/dead distinction for 4°C for 30 min. Following a washing step, CD3 APC-Cy7 (BD, New Jersey, USA) CD4 PE-Cy7 (BD, New Jersey, USA), CD19 PerCP-Cy5.5 (BD, New Jersey, USA), CD14 FITC (BD, New Jersey, USA), CD16 FITC (BD, New Jersey, USA), CD56 FITC (Miltenyi Biotec GmbH, Bergisch Gladbach, D), CD83 APC (BD, New Jersey, USA), CD11c PE (BD, New Jersey, USA) antibodies were added for 30 min at 4° C. After incubation, the cells were washed with FACS buffer (1% BSA, 0,1% NaN3 in 1x PBS) and divided into two tubes to perform extra- and intracelluar staining. The cells for extracellar staining were directly stained with CD83 APC (BD, New Jersey, USA) antibody for 30 min at 4°C and measured afterwards. For intracellular staining, cells were previously fixated with 100 μL 1× eBioscience Fixation solution (Thermo Fisher Scientific, Waltham, Massachusetts, USA) for 20 min at room temperature kept in the dark. The cells were then permeabilized in 500 μl eBioscience Permabilization Buffer (Thermo Fisher Scientific, Waltham, Massachusetts, USA) for 5 min at 4°C and afterwards stained with CD83 APC (BD, New Jersey, USA) antibody for 30 min at 4°C.

Flow cytometric measurement was performed using the BD FACSCanto™ Clinical Flow Cytometry system. Data were analyzed in the FlowJo software (FlowJo V.10.1, FlowJo, LLC, Ashland, Oregon, USA). Samples were treated and measured in duplicates.

For flow cytometric analysis of EVs, Vybrant™ Dio Cell-Labeling Solution (Thermo Fisher Scientific, Waltham, Massachusetts, USA) was added to supernatants 15 min prior to collection of supernatants. Isolated EVs were stained with CD9-APC (Miltenyi Biotec GmbH, Bergisch Gladbach, D), CD63-PE-Cy7 (BD, New Jersey, USA) and CD83-PE (BD, New Jersey, USA) antibodies for 30 min at 4°C in the dark. PE Mouse IgG1 κ Isotype Control was used as CD83-negative control and Cytokeratin 7-APC (Miltenyi Biotec GmbH, Bergisch Gladbach, D) antibody was used as CD9 isotype control. Size reference of EVs was performed using Flow Cytometry Sub-Micron Size Reference Kit (Thermo Fisher Scientific, Waltham, Massachusetts, USA). Flow cytometric analysis was performed on the Attune NxT with Attune™ NxT Small Particle Side-Scatter Filter. Data were analyzed with FlowJo software (FlowJo V.10.10, FlowJo, LLC, Ashland, Oregon, USA).

### 2.6 ELISA

Cell culture supernatants were analyzed with a R&D DuoSet ELISA kit human sCD83 (DY2044-05) (R&D Systems, Minneapolis, USA) following manufacturer’s instructions. Samples were tested in duplicates.

### 2.7 Multiplex assay

CD3^+^ and CD19^+^ supernatants were characterized using an 8-plex Magnetic Bead Kit (MMP-2, MMP-7, MMP-9, MMP-13, TIMP-1, PD-L1, PD-L2, TGF-β; ThermoFisher Scientific, Schwerte, Germany) for detection in the Bio-Plex™ 200 System (Bio-Rad Laboratories, Hercules, USA). Analyte concentrations were determined in duplicates, the means are shown (pg/mL).

### 2.8 RNA extraction and quantitative real-time PCR

Cells were harvested in TRIzol^®^ (ThermoFisher Scientific, Waltham, USA). Chloroform (> 99%; Chemsolute, Roskilde, Denmark) was added and phases were separated during 15 min centrifugation at 10,000 × *g* and 4°C. RNA was precipitated with Isopropanol (> 99%; Chemsolute, Roskilde, Denmark) for 1 h at –20°C, while RNA visibility was improved using Glyco Blue™ (ThermoFisher Scientific, Waltham, USA). After 20 min centrifugation at 10,000 × *g* and 4°C, RNA was washed with Ethanol (96%; Walter CMP, Kiel, Germany) centrifuged again for 6 min at 10,000 × *g* and 4°C and dried carefully. RNA was resupended in DEPC water. For reverse transcription in the PeqSTAR thermocycler (PEQLAB, Erlangen, Germany), the High-Capacity cDNA Reverse Transcription Kit with RNase Inhibitor (ThermoFisher Scientific, Waltham, USA) was used according to manufacturer’s instructions. Quantitative Real-Time PCR (qPCR) was performed in the 7300 Real Time PCR thermocycler (ThermoFisher Scientific, Waltham, USA) using the Power SYBR(R) Green PCR Master Mix (ThermoFisher Scientific, Waltham, USA) and primers flanking *CD83* or *ACTB*, respectively. The cycling program was adjusted according to manufacturer’s protocol with 2 min at 50°C before pre-heating at 95°C for 10 min, followed by 41 PCR cycles with a denaturing temperature of 95°C for 15 s and annealing and elongation at 60°C for 1 min. The final PCR cycle was terminized with 95°C for 15 s. Relative quantification values for *CD83* mRNA expression were calculated *via* comparative threshold cycle method (2^-ΔΔCt^) using *ACTB* (Beta-Actin) as an internal reference. The used oligonucleotides (ThermoFisher Scientific, Waltham, USA) are listed in **Supplementary Table 2**. The means of quadruplicates are shown for six biological replicates. Primer sequences are shown in **Supplementary Table 1**.

### 2.9 *In silico* cleavage analysis

A cleavage prediction analysis with Procleave (procleave.erc.monash.edu/Procleave_crf/webserver.html) was performed on the human CD83 molecule (GenBank: AAH30830.1) (41). Candidates targeting the extracellular domain of CD83 (residues 20-144) were identified and selected for further analysis when the predictive cleavage score was higher than 0.7 (see **Supplementary Table 2**).

### 2.10 Statistical analysis

Data represent the average of biological replicates obtained in independent experiments as described in the figure legends. Statistical analysis was performed using the GraphPad Prism 9 software. Data were tested for normal distribution before analysis. Statistical significance was determined using a one-way ANOVA with Dunnett’s post-hoc analysis and is defined as p < 0.05 (*), p < 0.01 (**), p < 0.001 (***), or p < 0.0001 (****).

## 3 Results

### 3.1 LPS increases CD83 expression in decidual leukocytes

**Figure 1:**
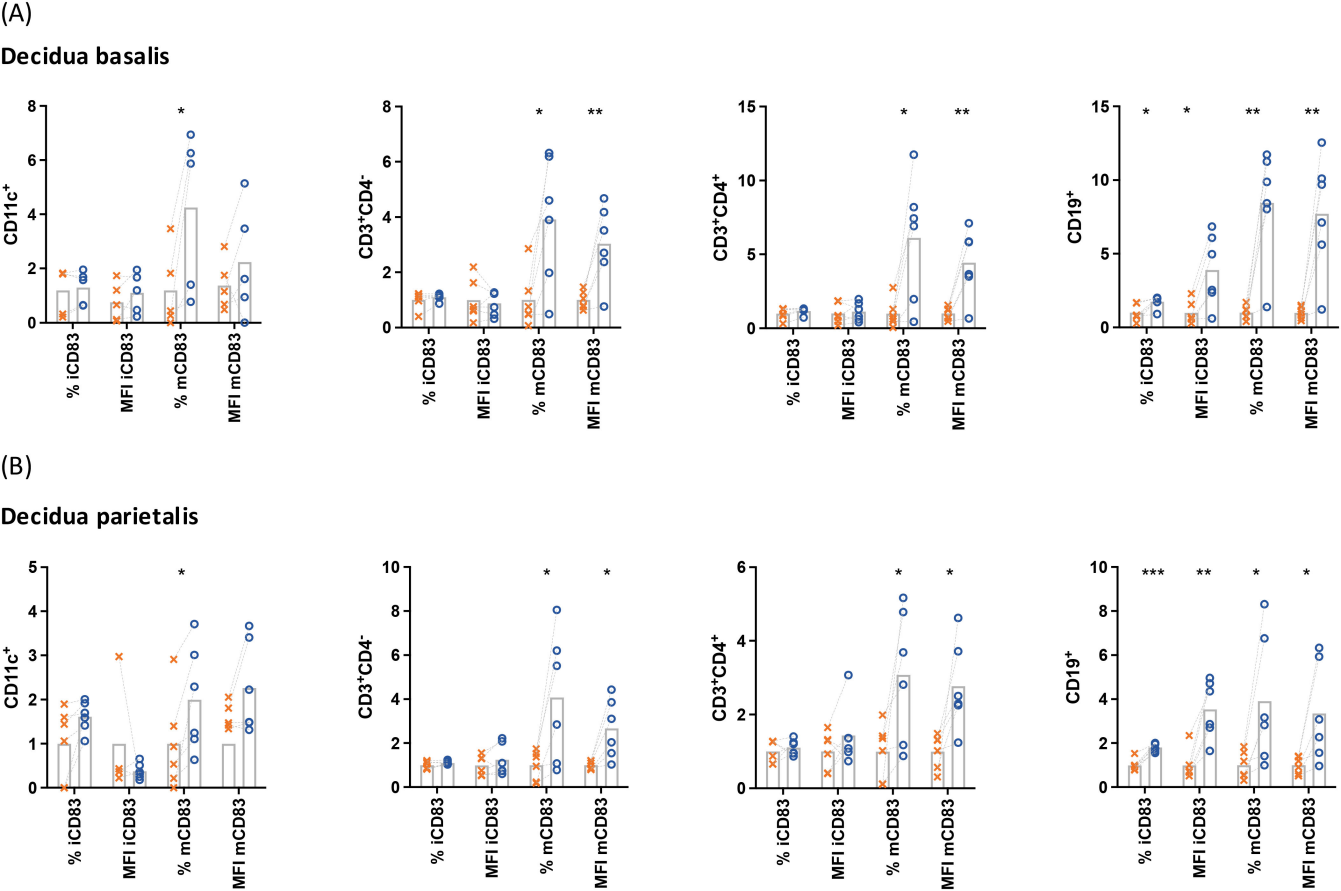
Mononuclear cells were isolated from the *decidua basalis* **(A)** or *decidua parietalis* **(B)** of term placentae, cultured in the presence (○) or absence (✕) of 10 µg/mL LPS, 50 ng/mL PMA, 500 ng/mL ionomycin and characterized by flow cytometry. Data were normalized to the respective mean of the control, analyzed by paired *t*-test, and presented as mean (n = 5). *p < 0.05; **p < 0.01; ***p < 0.001. MFI, mean fluorescence intensity; i, intracellular; m, membrane-bound (extracellular). Gating strategies are shown in **Supplementary Figure 1**.

To evaluate the involvement of CD83 in the tolerogenic responses of the human decidua, *decidua basalis* and *decidua parietalis* mononuclear cells were treated with LPS and the intracellular and extracellular expression of CD83 was evaluated by flow cytometry (**Figure 1**). After LPS treatment, a significant increase of CD83 expression could be detected in cells from *decidua basalis* and *decidua parietalis* intracellularly (iCD83) as well as extracellularly (mCD83). Particularly B lymphocytes experienced an upregulation in both extra and intracellular compartments, while T cells and DCs predominantly showed changes in mCD83 expression. These data indicate that leukocytes from both decidual compartments react to bacterial LPS via induction of CD83 expression.

### 3.2 CD83 is preferentially increased in B lymphocytes after LPS treatment

**Figure 2:**
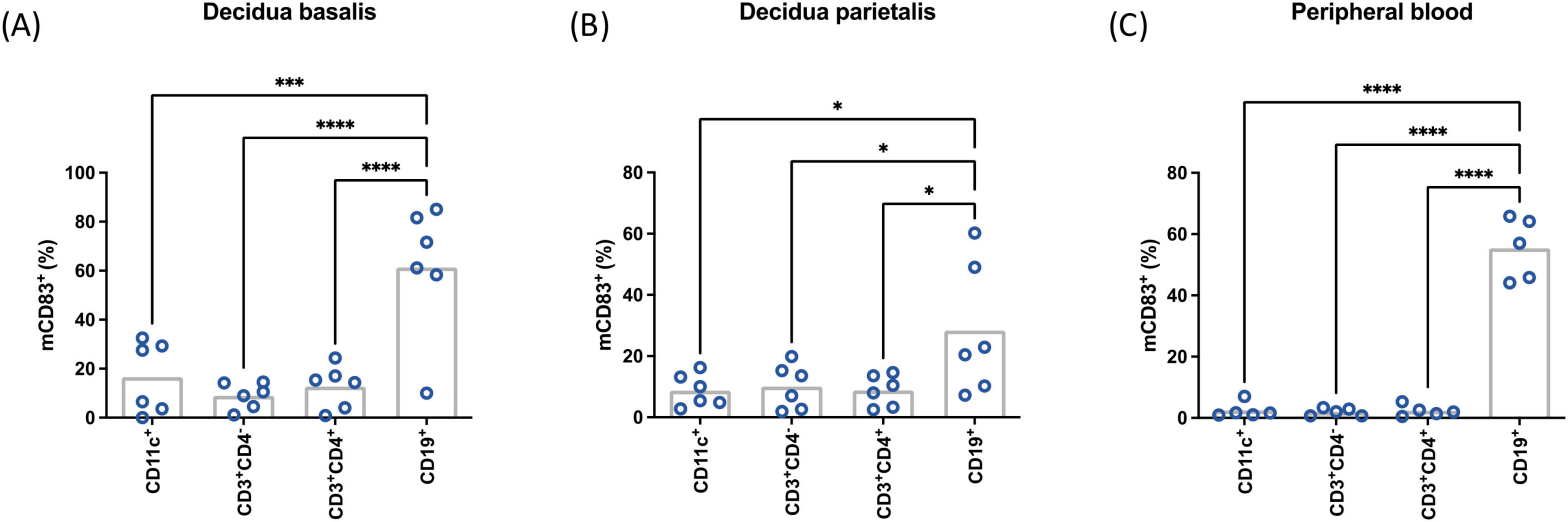
Mononuclear cells were isolated from term placental *decidua basalis* **(A)**, *decidua parietalis* **(B)**, or peripheral blood **(C)** stimulated with 10 µg/mL LPS, 50 ng/mL PMA, 500 ng/mL ionomycin, and characterized by flow cytometry. Data were analyzed by paired ANOVA with Tukey post-test **(A)** and presented as mean (a,b: n = 6; c: n = 5). *p < 0.05; ***p < 0.001; ***p < 0.0001. MFI, mean fluorescence intensity. Gating strategies are shown in **Supplementary Figure 1**.

In order to identify immune cell populations that preferentially express mCD83 upon LPS stimulation, cells from *decidua basalis*, *decidua parietalis* and peripheral blood from female non-pregnant donors were analyzed (**Figure 2**). Similar to our previous observations in multiple lymphoid organs in mice, *decidua basalis* B cells were the cells expressing mCD83^+^ (accounting for approximately 60% of B cells) most distinctly after LPS stimulation. Though less prominent, a similar pattern could be observed in *decidua parietalis*, while peripheral blood cells showed a clear predominance of CD83 expression in B cells among all studied leukocytes. These results suggest that B cells are the main source of sCD83 at the fetomaternal interface, especially in *decidua basalis* as compared to *decidua parietalis*.

### 3.3 Decidual leukocytes have a low capacity for sCD83 release

**Figure 3:**
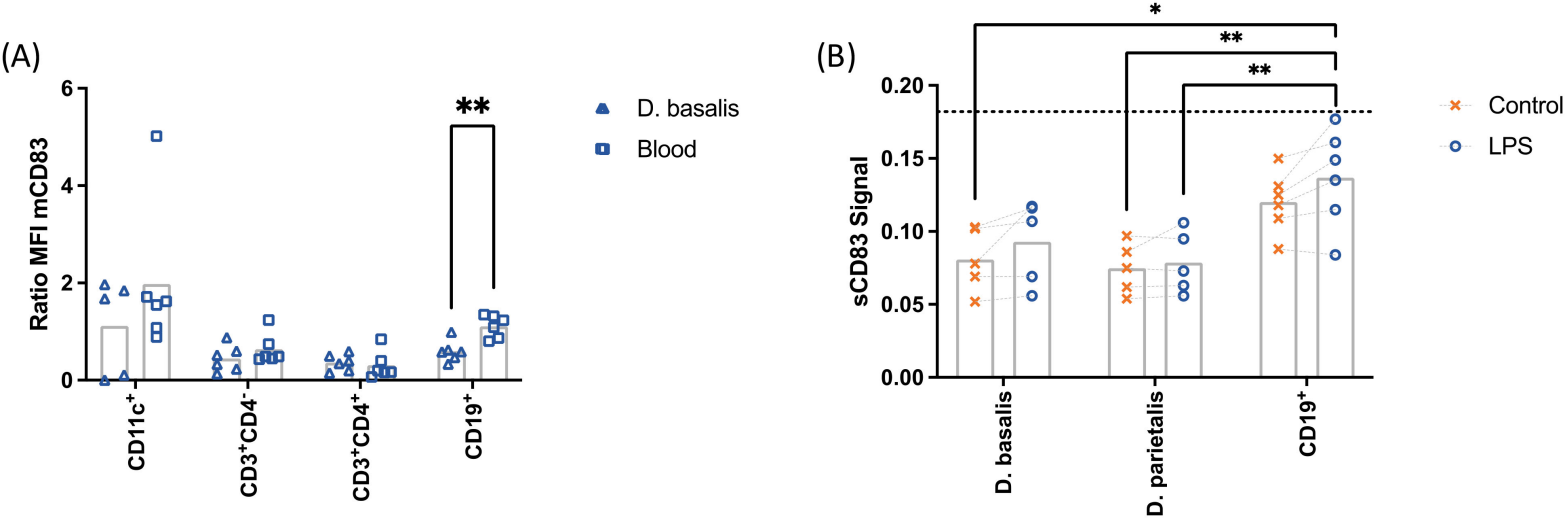
Mononuclear cells from *decidua basalis* (△) or peripheral blood (□) were isolated. Expression of mCD83 in CD11c^+^, CD3^+^CD4^-^, CD3^+^CD4^+^ and CD19^+^ cells after stimulation with 10 µg/mL LPS, 50 ng/mL PMA, 500 ng/mL ionomycin was compared using flow cytometry (ratio = stimulated:control; **A**). Mononuclear cells from *decidua parietalis* or *decidua basalis* and B cells from *decidua basalis* were isolated from term placentae and cultured in the presence (○) or absence (✕) of 10 µg/mL LPS, 50 ng/mL PMA, 500 ng/mL ionomycin. Cell culture supernatants were analyzed by ELISA **(B)**. The dotted line represents the mean of the lowest standard. Data were analyzed by unpaired *t-*test **(A)** or ANOVA with Tukey **(B)** post-test and are presented as mean (a: n = 6; b: n = 4-6). *p < 0.05; **p < 0.01. Gating strategies are shown in **Supplementary Figure 1**.

To evaluate the capacity of decidual leukocytes to release sCD83, the increment of mCD83 expression after LPS treatment was compared between cells from *decidua basalis* and peripheral blood from female non-pregnant donors (**Figure 3**). B cells from the *decidua basalis* showed a reduced capacity to increase mCD83 expression after LPS stimulation as compared to peripheral blood leukocytes. No significant differences were observed in T cells and DCs.

Similar results were obtained for the analysis of sCD83 release. Conditioned media of the LPS-treated decidual leukocytes was analyzed by ELISA. The levels of sCD83 were detected below the lowest assay standard and therefore could not be quantified. Despite this, the obtained data was further analyzed to get insights into the relevance of decidual B lymphocytes on sCD83 release. The detected levels of sCD83 released by B cells isolated from *decidua basalis* were higher than in leukocytes from *decidua basalis* and *decidua parietalis*. Overall, these data suggest a local regulation of sCD83 release.

### 3.4 Pregnancy-related molecules modulate LPS-induced mCD83 expression and sCD83 release

**Figure 4:**
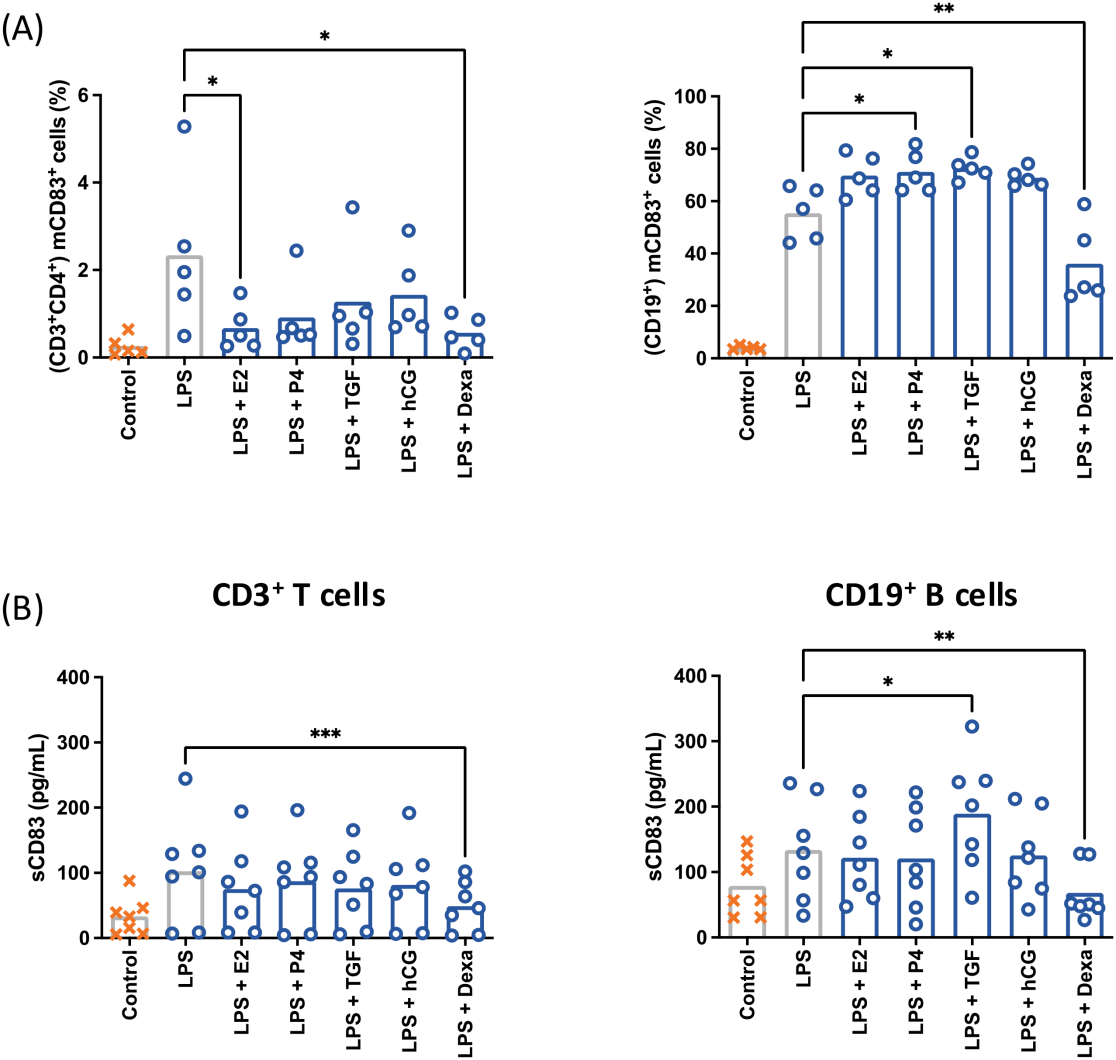
CD3^+^CD4^+^ T cells and CD19^+^ B cells from peripheral blood were characterized by flow cytometry after treatment with pregnancy-related factors **(A)**. E2: estradiol (2 ng/mL), P4: progesterone (30 ng/mL), TGFβ: TGF-β1 (2 ng/mL), hCG (10 IU/mL) Dexa, dexamethasone (40 ng/mL). CD3^+^CD4^+^ and CD19^+^ cells were isolated by magnetic separation and stimulated with 10 µg/mL LPS, 50 ng/mL PMA, 500 ng/mL ionomycin (○) in the presence of pregnancy-related factors. The supernatant of treated cells was analyzed by ELISA **(B)**. Data were analyzed by paired ANOVA with Dunnett post-test and are depicted as mean (a: n = 5; b: n = 7). *p < 0.05; **p < 0.01; ***p < 0.001. Gating strategies are shown in **Supplementary Figure 2**.

The capacity of pregnancy-related molecules to modulate LPS-induced mCD83 expression and sCD83 release was analyzed by flow cytometry and ELISA (**Figure 4**). T cells showed a reduction of mCD83^+^ cells after treatment with estradiol and dexamethasone, as well as a reduction of sCD83 after treatment with solely dexamethasone. Progesterone, TGF-β1 and hCG lead to a determinable though statistically not significant reduction of mCD83 expression and sCD83 release.

CD83-expressing B cells were increased after treatment with progesterone and TGF-β1 but reduced after dexamethasone treatment. There was also a significant increase in sCD83 release after treatment with TGF-β1 and a significant reduction of sCD83 release after dexamethasone treatment. Estradiol and hCG treatment induced a statistically non-significant increase of mCD83 expression, but had no effect on sCD83 release.

These data indicate that pregnancy-associated factors are able to modulate mCD83 expression and sCD83 release in leukocytes.

### 3.5 LPS induced increase of CD83 attributable to sCD83 and not EV-bound mCD83

**Figure 5:**
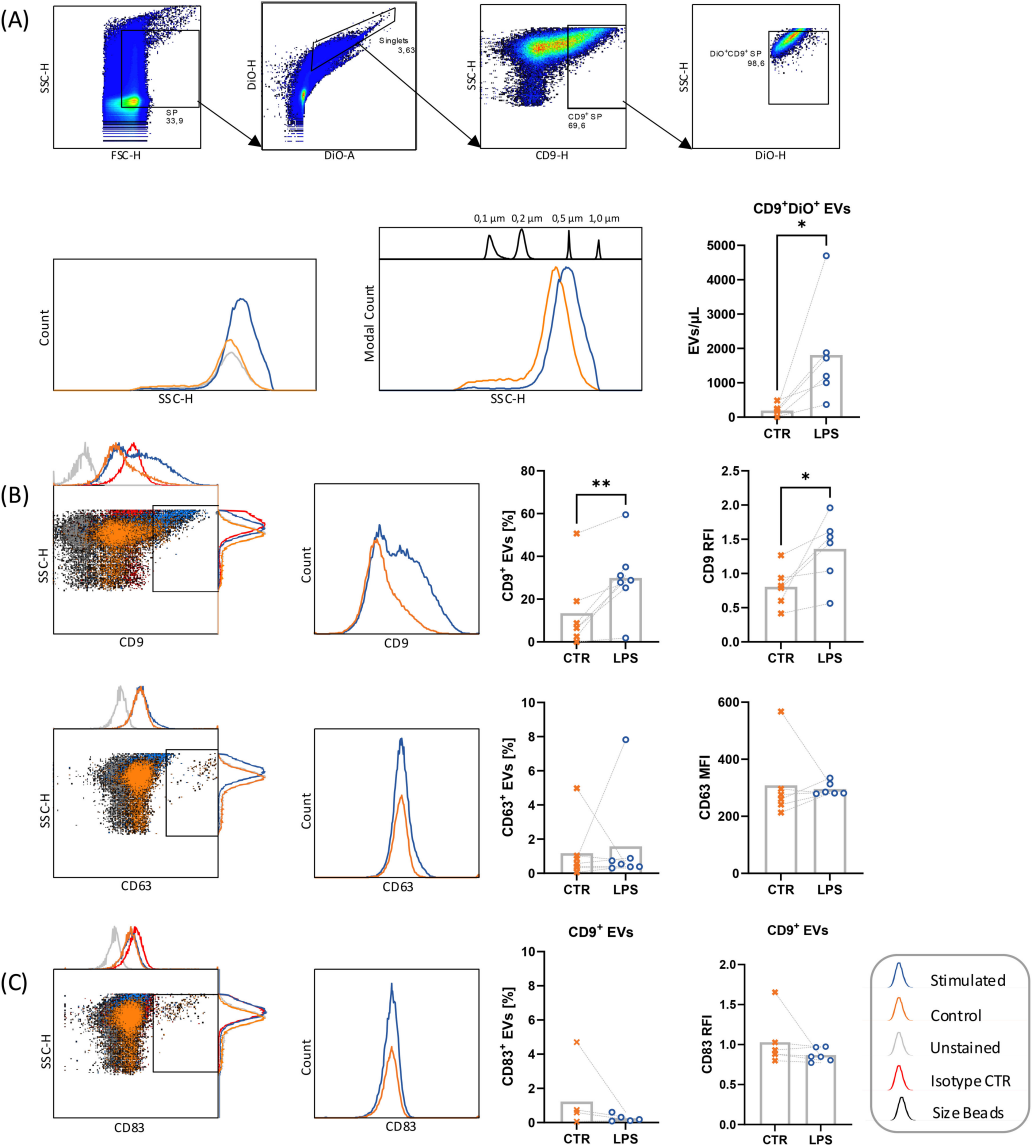
Flow cytometric analysis of EVs from PBMC supernatants before (CTR, ✕) and after stimulation with 10 µg/mL LPS, 50 ng/mL PMA, 500 ng/mL ionomycin (LPS, ○). DiO^+^ small particles (SP) of stimulated PBMC were analyzed by flow cytometry after EV isolation using Invitrogen Total Exosome Isolation reagent. **(A)** Small particles were gated for single events according to DiO-H to DiO-A ratio. Single events were first gated for CD9^+^ SP, followed by gating for DiO-stained SP, excluding high-SSC DiO-dye crystals. Henceforth, DiO^+^CD9^+^ SP were called extracellular vesicles (EVs). EVs were cross-checked with size reference beads. **(B)** DiO^+^ stained single events were investigated for their CD9 and CD63 expression. CD9 RFI calculated from CD9 MFI of sample in relation to isotype control. **(C)** DiO^+^CD9^+^ EVs were investigated for CD83 expression. RFI calculated from CD83 MFI of sample in relation to isotype control. Data were analyzed by paired *t-*test and are depicted as mean (n = 6). *p < 0.05; **p < 0.01. RFI, Relative Immunofluorescence.

The detection of sCD83 by ELISA in cell culture media of LPS-stimulated leukocytes could be attributed to the release of CD83 as a soluble protein or to the presence of the membrane-bound form in the surface of extracellular vesicles (EVs). To verify the latter, EVs were isolated from cell conditioned media and analyzed by flow cytometry (**Figure 5**) and ELISA (data not shown). Characterization of EVs was based on CD9- and CD63-expression on DiO-stained small particles, which were cross-checked by size reference beads (**Figure 5A**). We showed significantly higher number of isolated CD9^+^ exosomes from LPS stimulated PBMCs as compared to non-stimulated controls. This effect could be seen after 24 h as well as after 48 h stimulation with LPS, PMA and Ionomycin. Additionally, two populations of EVs were detected in unstimulated control samples, with one population encompassing exosomes up to 200 µm, and a second population of ectosomes in a size range of 200 – 500 µm. Upon LPS stimulation, samples showed less exosomes < 200 µm diameter, but a significant increase of EVs mostly within the size range of 200 – 1000 µm. EVs of LPS-stimulated samples additionally showed a significant increase of CD9-expressing DiO^+^ EVs and CD9 RFI (CD9 MFI of sample/CD9 MFI of isotype control) (**Figure 5B**). Interestingly, CD63-expressing DiO^+^ EVs were slightly increased in LPS-stimulated samples, yet CD63 MFI stagnated and tended to be decreased, implying a shift to CD9^+^CD63^-^ ectosome- over CD9^+^CD63^+^ exosome-production.

Considering the low CD63 expression upon LPS stimulation, mCD83 expression was also analyzed on DiO^+^CD9^+^ EVs (**Figure 5C**). Despite the increase in the numbers of DiO^+^CD9^+^ EVs after LPS stimulation, low mCD83 expression could be detected, which was even slightly decreased after LPS stimulation. Similarly, when magnetically purified CD63^+^, CD9^+^ and CD81^+^ EVs from 24 h LPS stimulated mononuclear cells were analyzed by ELISA, no CD83 could be detected.

These results indicate that the higher expression of CD83 in the culture media of LPS-stimulated mononuclear cells can be attributed to an increased release of sCD83 and not to an increase in the number of CD83^+^ EVs.

### 3.6 Pregnancy-related molecules regulate CD83-targeting protease MMP-7

**Figure 6:**
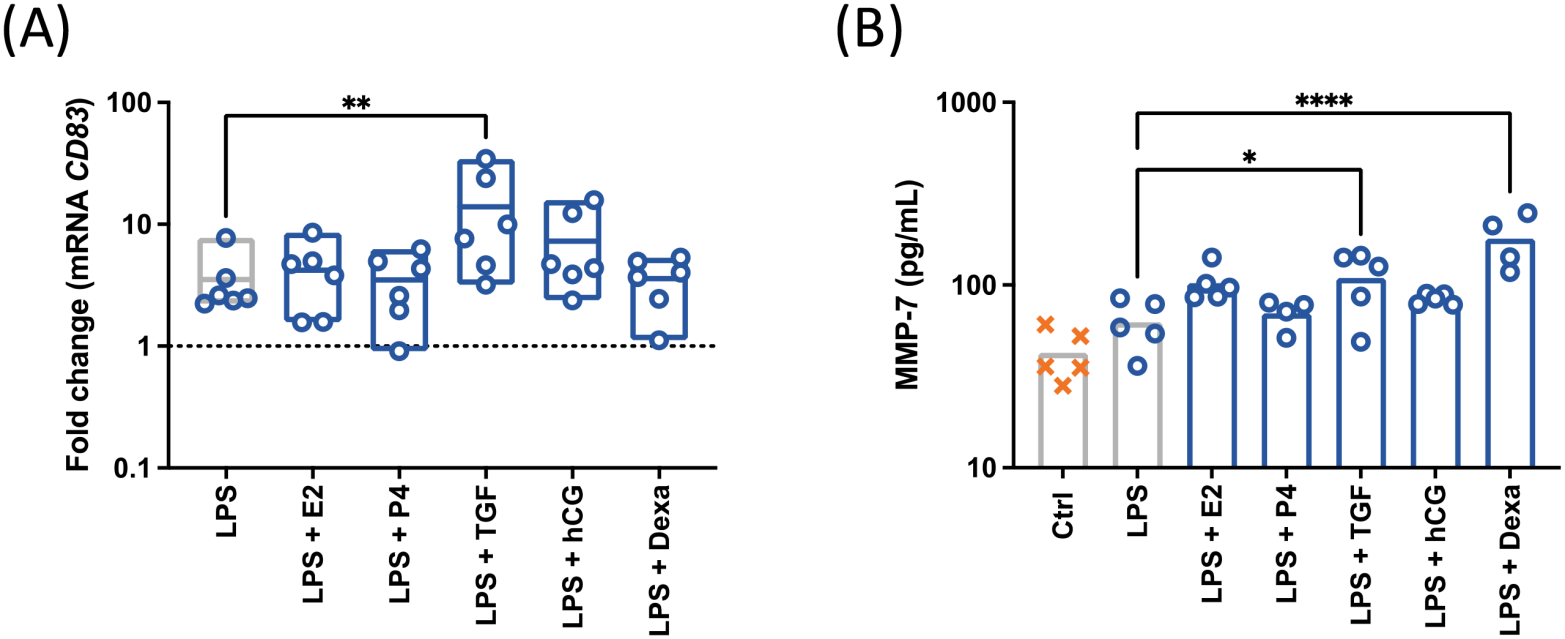
CD19^+^ cells were isolated by magnetic separation and stimulated with 10 µg/mL LPS, 50 ng/mL PMA, 500 ng/mL ionomycin (○) in the presence of pregnancy-related factors. E2, estradiol (2 ng/mL), P4: progesterone (30 ng/mL), TGFβ, TGF-β1 (2 ng/mL), hCG (10 IU/mL) Dexa, dexamethasone (40 ng/mL). The expression of *CD83* was assessed by qPCR **(A)** and the secretion of MMP-7 was analyzed by ELISA **(B)**. Data were analyzed by paired ANOVA with Dunnett post-test and are depicted as mean (n = 6). *p < 0.05; **p < 0.01; ****p < 0.0001.

As pregnancy-related factors modulated both mCD83 expression and sCD83 release, a possible induction of *CD83* gene transcription was evaluated (**Figure 6A**). First, a promotion of *CD83* mRNA expression in LPS-treated cells was observed after of TGF-β1 treatment.

Then, as the increase or reduction of sCD83 release could be caused by enhanced cleavage of the membrane-bound form or its degradation, respectively, modulations in protease activity were considered (**Figure 6B**). Based on cleavage prediction analysis of the extracellular structure of CD83 (Proclave), the highest predictive cleavage values (>0,700) were detected for MMP-2 (0,924), MMP-7 (0,721) and MMP-9 (0,703). As observed in our previous work, we identified TIMP-1 as a regulator of mCD83 expression. The MMPs targeted by TIMP1 (MMP-7 and MMP-9-) were therefore quantified in the conditioned media of treated cells. Both TGF-β1 and dexamethasone induced an increase of MMP-7 in supernatants of LPS-treated cells. Regarding *MMP9* and *TIMP1* mRNA levels, no significant modulation by pregnancy-related molecules could be observed (**Supplementary Figure**).

These data suggests that TGF-β1 and dexamethasone promote a MMP-7-mediated degradation of mCD83 which is overcome by an increment of gene transcription in the case of TGF-β1 (**Supplementary Figure**).

## 4 Discussion

The maternal immune responses against infections has to adapt in order to minimize the risk of compromising fetal tolerance during pregnancy (2, 42–44). The relevance of sCD83 in modulating excessive pro-inflammatory immune responses has been previously demonstrated (31, 32, 34–37, 45–48). Its role for pregnancy well-being however, has not been studied extensively. Murine studies suggest its relevance in response to inflammatory stimuli derived from bacterial components as LPS (15, 17). Human studies of CD83 during pregnancy are sparse but indicate a systemic control of sCD83 release (40).

In this work, we were able to characterize mCD83-expressing cells in *decidua basalis* and *decidua parietalis* of human pregnancies at term. Additionally, CD83-staining was performed after fixation and permeabilization of the cells (iCD83), providing data on the intracellular expression of the CD83 molecule. Although the extracellular expression of CD83 was not blocked before the intracellular staining, the iCD83 expression was significantly higher than the mCD83 staining. When the cells were stimulated, the increase of mCD83 expression was approximately 5 times higher than the iCD83 staining (control mCD83/iCD83 versus stimulated mCD83/iCD83 between 4.8 and 5.2). For this reason, we attribute the changes in CD83 mainly to a promotion of mCD83 expression rather than an increased *de novo* synthesis. However, further studies could address this in more detail.

In both *decidua basalis* and *decidua parietalis*, treatment with bacterial LPS led to an increased expression of mCD83 in dendritic cells, T cells and B cells. After this increment, mCD83^+^ cells were observed specially within the B cell subset. This observation was particularly distinctive in leukocytes isolated from the *decidua basalis*. Previous studies have described multiple differences between the composition of leukocytes isolated from *decidua basalis* and *decidua parietalis* (49). These differences might be related to the microenvironmental characteristics of each compartment. In particular, *decidua basalis* is exposed to higher levels of oxygen than *decidua parietalis* and stands in closer contact to other immunoregulatory cells such as decidual stroma cells and trophoblasts. Furthermore, the decidual leukocyte composition is influenced by blood derived immune cells.

In our study, B cells from *decidua basalis* contained a higher percentage of CD83^+^ cells than peripheral blood B cells after LPS Stimulation. However, the relative increase upon stimulation with LPS was lower in *decidua basalis* B cells. After evaluating the sCD83 secretory capacity, only low levels of sCD83 could be detected in the supernatants of cultured decidual mononuclear cells, hindering the quantification and further evaluation of the treatment with certainty. As similar number of B cells were stimulated *in vitro*, the data point to an individual decidual mCD83^+^ B cell compartment. In this context it is possible that decidual B cells experience a different regulatory environment than those in the periphery. There are several decidual immune cells with a specific decidual phenotype, which is partially determined by the role of hormones and other factors present at the fetomaternal interface (44, 50–54). As term deciduae were analyzed during this work, a regulated sCD83 release to facilitate inflammatory mechanisms during parturition cannot be discarded. However, considering the use of enzymatic digestion during decidual lymphocyte isolation and its possible influence on protease activity, this observation should be seen with caution.

Since peripheral B cells were obtained from blood from non-pregnant female donors, a modulation of CD83 expression by pregnancy-related factors was only evaluated *in vitro*. There was no effect of pregnancy-related hormones on baseline mCD83 expression (not shown). However, they clearly modulated the capacity of B-cells to express mCD83 and release sCD83 in the presence of pro-inflammatory triggers. These data support our previous observation from mouse models, where B cells acquired an enhanced capacity to express mCD83 and release sCD83 during pregnancy (15, 17).

In spite of the hormone-based increase of mCD83/sCD83 on stimulated leukocytes, it is remarkable that the release of sCD83 from decidual mononuclear cells was considerably low. As the release of sCD83 was LPS-triggered, we postulate that sCD83-mediated regulation predominantly modulates systemically, but less effectively in confrontation to local infections. This might result advantageous during infections during pregnancy, as immune responses distant from the fetomaternal interface will be accompanied by a boost of sCD83 release, contributing to pregnancy maintenance. Our data, on the other hand, do not support a local sCD83-mediated regulation of immune responses towards infections at the fetomaternal interface. However, a sCD83-dependent regulation at the fetomaternal interface might occur though the influence of systemic sCD83 or by infiltrating leukocytes. Alternatively, this might represent an advantage to prioritize inflammation to foster immune responses against local infections.

It remains unknow which factors account for the differential sCD83 secretory capacity between peripheral and decidual B cells. Differences in B cell phenotype between both compartments have been observed previously and include a higher CD83 expression on resting endometrial B cells (39). However, in this and in our study, B cell subpopulations were not analyzed in detail. In peripheral blood and decidua, B cells represent a heterogeneous cell type which can be subdivided in two major groups: B1 (B1-a, B1-b) and B2 subsets (Marginal zone and follicular zone B cells, plasma cells, etc) (55, 56). Murine and human studies have shown that pregnancy induces changes in the development and function of several B cell subtypes (5, 56–63). Among these adaptations, an increased development of marginal zone B cells over follicular zone B cells could be observed. Although the expression and relevance of CD83 on B2 development and function has been well investigated, less is known about its relevance of B1 B cell biology (21, 27, 64). Therefore, as the distribution of each subset varies between peripheral blood and decidua, it is possible that these differences reflect the CD83-behavior we observed on total CD19^+^ B cells (65, 66).

Our results indicate that sCD83 might protect pregnancy from systemic but not local infections. While local leukocytes have a reduced sCD83 secretion capacity, blood derived leukocytes remain a main source of sCD83, although not through the secretion of CD83^+^ EVs. To evaluate how pregnancy alters mCD83 and sCD83 release on peripheral leukocytes, we performed a series of *in vitro* experiments in the presence of hormones and further pregnancy-related factors.

Hormones exert regulatory roles shaping the dynamics of the immune system during the different stages of pregnancy. In general terms, hormones including E2, P4 and hCG promote tolerogenic responses that facilitate fetal tolerance and strengthen immune balance (67–70). In our experiments, an increment of CD83 expression could be observed after treatment with E2, P4 and hCG, but no considerable increment of sCD83 release was detected. This observation suggests that if mCD83 represents a source of sCD83, the membrane expression and the release of a soluble form might be independently modulated by hormones during pregnancy. Indeed, as we discussed in a previous work, the modulation of proteinases of the stromal tissue might exert an important role in the sCD83 release (17). This is a strong limitation in our work, as we evaluated the role of hormones in an *in vitro* set-up with peripheral blood mononuclear cells. A closer look into the modulation of proteolytic enzymes that target mCD83 has been discussed below. Nevertheless, it is still possible that the increase in CD83 expression on B cells enhances further tolerogenic adaptations, such as IL-10 release and the promotion marginal zone cell development (26–28).

TGF-β1 is an important modulator of immune cell function at the fetomaternal interface and its circulation increases during pregnancy (71, 72). TGF-β1 induces immunosuppressive regulatory T cells (Tregs), regulates the function of decidual NK cells and promotes M2-like macrophages (73). In our study, TGF-β1 showed a clear induction of CD83 production in mRNA level, membrane expression and release.

To evaluate the role of hormonal factors linked to late pregnancy and parturition, we analyzed the effects of glucocorticoids (dexamethasone) *in vitro*. The treatment led to a reduced mCD83 expression and sCD83 release from T cells and B cells. This could prevent sCD83 from interfering with the pro-inflammatory cascade occurring in the context of labor (74–77). Additionally, based on these data, sCD83 levels could be expected to be reduced in pregnant women experiencing high levels of stress, therefore high levels of cortisol. A possible role of stress/cortisol-mediated sCD83 reduction in preterm birth should be addressed in further studies (78).

As reported previously in mouse models, the bioavailability of mCD83 and subsequently sCD83 are likely to be subjects of hormone-dependent regulation of the proteolytic machinery (17). In an earlier work, we had found that negative regulation through the inhibitor of metalloproteinases TIMP-1 induces mCD83 expression and release, pointing to a proteinase-related appearance. The protease families ADAM and MMPs are modulated by TIMP-1 and strongly regulated during pregnancy (79). However, we had not found evidence of ADAM involvement in CD83 in our previous work and looked into MMPs closer in this study.

TIMP-1 is known to modulate MMP-1, MMP-3, MMP-7 and MMP-9 (80). After performing an *in silico* degradation of the external membrane segments of CD83, we found MMP-7 and MMP-9 to be feasible candidates for CD83 cleavage. Both enzymes displayed a cleavage “predictive score” higher than 0.7 (41). MMP-7 showed an inverse correlation to mCD83 expression and sCD83 release after treatment with dexamethasone, suggesting an MMP-7 mediated degradation of mCD83. However, after TGF-β1 treatment, increased levels of mCD83 and sCD83 were observed in the presence of high levels of MMP-7. This phenomenon could be explained through the compensatory upregulation of *CD83* mRNA transcription. A summary of the relative effects of hormone treatment on mCD83, sCD83, *CD83* mRNA and MMP-7 levels is listed in the supplements (**Supplementary Figure 3**).

The results of this study encourage a deeper characterization of mCD83/sCD83 during pregnancy. While the most relevant data of this study relies on *in vitro* observations, further studies could engage a larger number of patients to better reflect *in vivo* data. This will be particularly interesting during the different stages of pregnancy, which is accompanied by systemic changes in the hormonal levels. Furthermore, this study did not include samples from deciduae after spontaneous labor, which could depict further regulations of CD83 during the pro-inflammatory process of parturition. The effect of hormones and further immune modulatory factors could be studied in non-pregnant women in more detail. Although we only studied samples from healthy women in reproductive age, data concerning hormonal intake is missing. Larger studies could evaluate the role of menopause, contraceptives or hormonal therapy on CD83 expression and also provide further interesting data, including stress levels, demographics, etc.

In this work, we showed that mCD83 expression and sCD83 release is induced by LPS treatment in the fetomaternal interface. However, the contribution of systemic sCD83 might be stronger and subjected to a modulation by pregnancy-related molecules. This regulation is associated with changes in the production of CD83 and the modulation of proteolytic activity. In summary, the regulation of mCD83 and sCD83 release fits the model of tolerogenic mechanisms that prevails during mid pregnancy but declines towards the end of pregnancy.

## Data Availability

The data presented in the study are deposited in the Figshare repository, DOI number 10.6084/m9.figshare.26947969.
