# Supplementary material for: Pregnancy-related factors induce immune tolerance through regulation of sCD83 release
Source: Front Immunol. 2024 Sep 12;15:1452879. doi: 10.3389/fimmu.2024.1452879 (PMC11424458; doi:10.3389/fimmu.2024.1452879)
Supplement: Supplementary file 1 [file DataSheet1.docx]

Supplementary Material

**Pregnancy‑related factors induce immune tolerance through regulation of sCD83 release.**

Pauline Krupa, Hannah Wein, Lea Sophie Zemmrich, Marek Zygmunt, Damián Oscar Muzzio*

Research laboratory, Department of Obstetrics and Gynecology, University Medicine Greifswald, Greifswald, Germany

*** Correspondence:**Damián Muzzio
damian.muzzio@med.uni‑greifswald.de

# Supplementary Figure 1

## Supplementary Figure 1.

Gating strategies for the identification and characterization of CD19^+^ B cells, CD3^+^CD4^+^ T helper cells (**a**) and (CD14^-^CD16^-^CD56^-^CD19^-^CD3^-^CD4^-^)CD11c^+^ dendritic cells (**b**) in *decidua basalis* (depicted), *decidua parietalis* and peripheral blood by flow cytometry. Overlapping histograms show the expression of CD83 in FMO (grey), controls (orange) and samples stimulated with LPS, PMA and ionomycin (blue). mCD83 = membrane-bound CD83. iCD83 = intracellular CD83.

# Figure 2 Supplementary

FMO

Control

Stimulated

Stimulated + TGF-β1

Lymphocytes

Lymphocytes

Lymphocytes

Lymphocytes

Lymphocytes

42,1

42,1

42,1

42.1

42.1

Single Cells F

Single Cells F

Single Cells F

Single Cells F

Single Cells

96,7

96,7

96,7

96,7

96.7

Single Cells S

Single Cells S

Single Cells S

Single Cells S

Single Cells

97,6

97,6

97,6

97,6

97.6

Cocktail neg

Cocktail neg

Cocktail neg

Cocktail neg

Cocktail-

75,6

75,6

75,6

75,6

75.6

CD19

CD19

CD19

CD19

CD19+

9,96

9,96

9,96

9,96

9.96

CD19neg

CD19neg

CD19neg

CD19neg

CD19-

89.9

CD3- , CD4-

58.5

CD4- T cells

CD4- T cells

CD4- T cells

CD4- T cells

CD4- T Cells

40,3

40,3

40,3

40,3

40.3

Q1

Q1

Q1

Q1

Q1

1,19

1,19

1,19

1,19

1.19

Q2

Q2

Q2

Q2

Q2

52,7

52,7

52,7

52,7

52.7

Th cells

Th cells

Th cells

Th cells

Th Cells

52,4

52,4

52,4

52,4

52.4

CD83+

CD83+

CD83+

CD83+

CD83+

54,7

54,7

54,7

54,7

54.7

CD83+

CD83+

CD83+

CD83+

CD83+

0,77

0,77

0,77

0,77

0.77

FSC-H

SSC-H

FSC-A

SSC-A

FSC-W

SSC-W

FSC-H

CD14CD16CD56FVD

mCD83

CD19

SSC-A

SSC-A

mCD83

CD3

CD4

SSC-A

## Supplementary Figure 2.

Gating strategies for the identification and characterization of CD19^+^ B cells, CD3^+^CD4^+^ T helper cells in peripheral blood by flow cytometry after stimulation with pregnancy related factors. Overlapping histograms show the expression of membrane-bound CD83 (mCD83) in FMO (grey), controls (orange), samples stimulated with LPS, PMA and ionomycin (blue), and samples stimulated with LPS, PMA, ionomycin and pregnancy related factors (light blue) (depicted: TGF-β1).

# Supplementary figure 3

## Supplementary Figure 3.

CD19^+^ cells were isolated by magnetic separation and stimulated with LPS, PMA, and ionomycin in the presence of pregnancy-related factors. E2: estradiol (2 ng/mL), P4: progesterone (30 ng/mL), TGFβ: TGF-β1 (2 ng/mL), hCG (10 IU/mL) Dexa: dexamethasone (40 ng/mL). The secretion of MMP‑9 and TIMP‑1 was analyzed by ELISA **(a)**. Data were analyzed by paired ANOVA with Dunnett post-test and are depicted as mean ± SEM (n = 5). *p < 0.05; **p < 0.01; ***p < 0.001. The expression of *CD83* and mCD83, and the release of sCD83 and MMP‑7 are shown simultaneously to depict the individual relative effects of pregnancy-related factors **(b)**.

## Supplementary table 1. Donor information.

|  | Womens’ age | Week of gestation |
| --- | --- | --- |
| 1 | 26 | 39+1 |
| 2 | 22 | 39+0 |
| 3 | 42 | 39+3 |
| 4 | 28 | 40+5 |
| 5 | 41 | 38+6 |
| 6 | 31 | 39+1 |
| Mean ± SD | 31.8 ± 7.5 | 39.4 ± 0.6 |

## Supplementary table 2. Primer Sequences.

| Gene | Primer | 5’-3’ Sequence | Product length |
| --- | --- | --- | --- |
| *ACTB* | forward | CCTGGCACCCAGCACAAT | 70 bp |
|  | reverse | GCCGATCCACACGGAGTACT |  |
| *CD83* | forward | AAACAGCATCCTCCTTCCCAG | 127 bp |
|  | reverse | AGCAGAAACCCTCCCTCCAT |  |

## Supplementary table 3. Predicted protease-specific cleavage sites on human CD83.

| **Rank** | **Position** | **P4-P4' Site** | **N-fragment** | **C-fragment** | **Score** | **Family** |  |
| --- | --- | --- | --- | --- | --- | --- | --- |
|  |  |  |  |  |  |  |  |
| MMP-1 | | | | | | |  |
| 1 | 151 | LLLA†LVIF | 17.94 kDa | 6.46 kDa | 0.576 | M10.001 |  |
|  |  |  |  |  |  |  |  |
| MMP-2 | | | | | | |  |
| 1 | 168 | KFAR†LQSI | 19.98 kDa | 4.41 kDa | 0.946 | M10.003 |  |
| **2** | **118** | **GQRN†LSGK** | **13.99 kDa** | **10.40 kDa** | **0.924** | **M10.003** | ### |
| 3 | 196 | KHLG†LVTP | 23.28 kDa | 1.11 kDa | 0.719 | M10.003 |  |
| **4** | **92** | **RPYS†LKIR** | **10.84 kDa** | **13.55 kDa** | **0.677** | **M10.003** | # |
| 5 | 145 | YRAE†IVLL | 17.31 kDa | 7.08 kDa | 0.567 | M10.003 |  |
| **6** | **47** | **VPYT†VSWV** | **5.04 kDa** | **19.35 kDa** | **0.51** | **M10.003** |  |
|  |  |  |  |  |  |  |  |
| MMP-3 | | | | | | |  |
| **1** | **133** | **CPAQ†RKEE** | **15.75 kDa** | **8.64 kDa** | **0.669** | **M10.005** | # |
| **2** | **92** | **RPYS†LKIR** | **10.84 kDa** | **13.55 kDa** | **0.523** | **M10.005** |  |
|  |  |  |  |  |  |  |  |
| MMP-7 | | | | | | |  |
| **1** | **92** | **RPYS†LKIR** | **10.84 kDa** | **13.55 kDa** | **0.721** | **M10.008** | ## |
| 2 | 16 | CAYS†LAPA | 1.75 kDa | 22.64 kDa | 0.621 | M10.008 |  |
| 3 | 185 | ERAF†LPVT | 22.02 kDa | 2.37 kDa | 0.619 | M10.008 |  |
| 4 | 168 | KFAR†LQSI | 19.98 kDa | 4.41 kDa | 0.595 | M10.008 |  |
| 5 | 196 | KHLG†LVTP | 23.28 kDa | 1.11 kDa | 0.595 | M10.008 |  |
| **6** | **109** | **YRCT†LQDP** | **12.85 kDa** | **11.54 kDa** | **0.536** | **M10.008** |  |
|  |  |  |  |  |  |  |  |
| MMP-8 | | | | | | |  |
| **1** | **92** | **RPYS†LKIR** | **10.84 kDa** | **13.55 kDa** | **0.533** | **M10.002** |  |
|  |  |  |  |  |  |  |  |
| MMP-9 | | | | | | |  |
| **1** | **118** | **GQRN†LSGK** | **13.99 kDa** | **10.40 kDa** | **0.703** | **M10.004** | ## |
| **2** | **92** | **RPYS†LKIR** | **10.84 kDa** | **13.55 kDa** | **0.604** | **M10.004** | # |
| **3** | **47** | **VPYT†VSWV** | **5.04 kDa** | **19.35 kDa** | **0.588** | **M10.004** |  |
| 4 | 5 | MSRG†LQLL | 0.54 kDa | 23.85 kDa | 0.508 | M10.004 |  |
|  |  |  |  |  |  |  |  |
| MMP-12 | | | | | | |  |
| **1** | **92** | **RPYS†LKIR** | **10.84 kDa** | **13.55 kDa** | **0.594** | **M10.009** |  |
| 2 | 156 | VIFY†LTLI | 18.57 kDa | 5.82 kDa | 0.505 | M10.009 |  |
| **3** | **78** | **HQKG†QNGS** | **9.16 kDa** | **15.23 kDa** | **0.503** | **M10.009** |  |
|  |  |  |  |  |  |  |  |
| MMP-13 | | | | | | |  |
| **1** | **78** | **HQKG†QNGS** | **9.16 kDa** | **15.23 kDa** | **0.518** | **M10.013** |  |
| **2** | **47** | **VPYT†VSWV** | **5.04 kDa** | **19.35 kDa** | **0.514** | **M10.013** |  |
|  |  |  |  |  |  |  |  |

Score #: > 0.6; ##: > 0.7; ###: > 0.9. Black: extracellular domain cleavage sites.
